# Long-Term Efficacy of Chlorhexidine Gel in Single-Crown Implant Rehabilitation: A Five-Year Follow-Up Study

**DOI:** 10.3390/dj11100228

**Published:** 2023-09-25

**Authors:** Gianmaria D’Addazio, Eugenio Manciocchi, Giuseppe Tafuri, Ruggero Schiavone, Giovanna Murmura, Luan Mavriqi, Bruna Sinjari, Sergio Caputi

**Affiliations:** 1 Unit of Prosthodontics, Department of Innovative Technologies in Medicine and Dentistry, University “G. d’Annunzio” of Chieti-Pescara, 66100 Chieti, Italy; gianmaria.daddazio@unich.it (G.D.); eugenio.manciocchi@unich.it (E.M.); giuseppe.tafuri@unich.it (G.T.); rugero91@gmail.com (R.S.); giovanna.murmura@unich.it (G.M.); scaputi@unich.it (S.C.); 2 Electron Microscopy Laboratory, University “G. d’Annunzio” of Chieti-Pescara, 66100 Chieti, Italy; 3 Department of Dentistry, Albanian University, 1001 Tirana, Albania; luanmavriqi@yahoo.com

**Keywords:** chlorhexidine gel, marginal bone loss, peri-implantitis, implant survival rate, implant decontamination, dental implant complication

## Abstract

Chlorhexidine digluconate (CHX) has shown the ability to significantly reduce inflammation and marginal bone loss (MBL) at the 1-year follow-up but limited data exist regarding its long-term efficacy in peri-implant stability. The objective was to compare the long-term effects (5 years of follow-up) of a placebo gel (16 patients in Group A) or a 0.20% CHX gel (15 patients in Group B) used at each previous surgical and prosthetic stage. Control visits were conducted in 2022, investigating the long-term effects by biological, radiological, and clinical evaluation. The data were statistically analyzed. The research achieved a 96.7% implant success rate over five years, but 41.9% of patients did not attend annual oral hygiene check-ups. The average MBL was 1.04 ± 0.39 mm, with no significant differences between the two groups. Notably, patients who attended regular periodontal check-ups experienced significantly less MBL than those who did not (*p* < 0.05). At five years, direct effects of CHX were absent, with both groups showing moderate bone loss. However, the results suggest that early disinfection could enhance both short- and long-term outcomes. In fact, patients with initial minor MBL due to use of CHX, preserve this advantage also after 5 years of follow-up. Additionally, the data underscore the importance of annual check-ups in early detection and management of biological complications.

## 1. Introduction

Dental implants, widely recognized as a trustworthy and predictable avenue for the restoration of missing teeth, largely owe their success to the process of osseointegration, which fosters implant integration with the patient’s newly formed bone. According to worldwide literature, the survival rate of implants over a span of 5 years is approximately 90–98%, while it is about 89–95% over 10 years [1,2,3,4].

However, despite its widespread use and reliability, dental implant treatments are not devoid of complications. Early factors may include the failure of osseointegration, postoperative infections, or a lack of primary stability. Meanwhile, late complications include marginal bone loss (MBL), peri-implantitis, and mechanical complication [5,6,7,8,9].

The research community has given considerable attention to MBL, given its critical role in ensuring both the functional and aesthetic outcomes of implant treatment [10,11]. Historically, during the first year of implant loading, some degree of peri-implant bone loss was frequently observed, which typically decreased in subsequent years. Albrektsson et al., in 1986 [11], cited MBL as one of the primary criteria in assessing implant success rate, defining MBL up to 1 mm within the first year of implant loading and an average annual MBL of 0.2 mm during the follow-up period as success criteria [11]. Various potential contributing factors have been discussed, including implant macro-geometry, the implant neck region, surface topography, surgical trauma, the platform switching concept, the quantity of peri-implant soft tissue, implant surgical technique, residual bone, and microgaps at the junction of the implant and abutment. The study of these aspects have allowed for a reduction in MBL over the years but without a total elimination [8,12,13,14]. The microgap has been implicated as a principal site for bacterial colonization, and thereby a significant contributor to MBL [15,16,17,18]. This microscopical space, usually lying between 10 and 135 μm, is the subject of ongoing research efforts aimed at reducing its size and improving the implant–abutment connection [15,17,18,19,20,21]. Even though the use of more advanced connection types has allowed for some reduction in the microgap size, no existing connection type has yet succeeded in eliminating this area of potential bacterial ingress during long-term clinical use [16,17]. The existing microgap could cause micromotions and bacterial penetration, leading to a peri-implant inflammatory reaction and subsequent bone loss [15,16,17]. A perpetual infiltrate of inflammatory cells at the fixture–abutment interface has been observed in animal studies, suggesting a defensive response of the host to bacterial invasion [18]. In order to reduce bacterial colonization during the surgical and postsurgical stages, rigid protocols have been established, often involving the use of local antiseptics such as chlorhexidine (CHX). Renowned for its plaque inhibitory capabilities and broad-spectrum antimicrobial activity, CHX is a critical adjunct in current clinical practices, including oral and implant surgery. However, its benefits were often limited by its short-term application [19,22]. A previous study investigated the efficacy of CHX gel used in all surgical and prosthetic phases of single-implant restoration, and yielded encouraging results [23]. Specifically, it was an in vivo randomized and controlled patient study that compared two groups. The test group used chlorhexidine during all phases, while the control group used a placebo. The results showed that the use of CHX led to a reduction in short-term (12-month) peri-implant bone resorption after implant placement, demonstrating that a strict disinfection protocol of the microgap zone was effective in maintaining bone [23]. Subsequently, in a second study by D’ercole et al. in 2020 on the same patients, microbiological and immunohistochemical investigations were performed, demonstrating a reduction in inflammatory responses and bacterial loads in patients treated with CHX [24].

Given the demonstrated short-term efficacy of CHX in reducing bacterial load and MBL [23], the objective of this experimental study was to monitor the long-term outcomes of the same patient group and analyze peri-implant bone stability after more than 5 years. The null hypothesis was that there would be no difference in MBL after five years of follow-up between the use of a CHX and placebo gel during the clinical and prosthetic phases. The primary objective was to evaluate MBL at the 5-year follow-up.

The secondary objectives were to:

- Correlate the MBL described in the first study [23] and evaluate its trend over time.
- Analyze biological complications during 5 years of follow-up.

In addition, an evaluation of mechanical complications was conducted to enhance the comprehensiveness of the analysis. This assessment, distinct from considerations related to biological factors, contributes to a more expansive viewpoint concerning the overall condition of dental implants during the 5-year follow-up period.

## 2. Materials and Methods

### 2.1. Study Design and Sample

This study represents the second part of a prospective, randomized, controlled, double-blind clinical trial designed according to the Helsinki Declaration protocol. The allocation ratio was 1:1. The study was approved on 23 July 2015 by the Interinstitutional Ethical Committee of the University of Chieti-Pescara, Chieti, Italy, committee report n. 14. All patients provided written informed consent for treatment and enrollment in the study. The study was registered on clinicaltrials.gov with registration number NCT03431766. The second part of the study concerned a 5-year follow-up of the same patients. Therefore, data related to these patients were monitored over time and analyzed.

The study adhered to the CONSORT statement for improving the quality of RCTs.

The inclusion and exclusion criteria were fully reported in the previous study [23]. A succinct summary of the study’s inclusion and exclusion criteria, surgical aspects, and prosthodontic considerations is presented below.

The study included participants aged 18–75 with good overall and oral health, who needed single-crown implant-supported restorations, with adequate soft and hard tissue thickness. Patients with poor oral hygiene, active periodontal disease, insufficient bone thickness, or who had undergone bone augmentation procedures were not included. Additionally, patients with immediate loading protocols, uncontrolled diabetes, immune disorders, or those who smoked or had bruxism were also excluded.

Thirty-four patients with no noteworthy medical history, 20 males and 14 females, all non-smokers, were recruited for single-implant placement and prosthetic rehabilitation (age range 29–75 years; mean age 52.28). Patients were enrolled from December 2015 to March 2017 and were treated at the Dental Clinic of the Department of Medical, Oral and Biotechnological Sciences of the “G. d’Annunzio” University of Chieti-Pescara, Italy. Patients were randomly divided into Group A (control) (placebo gel (Placebo, Polifarma Wellness Srl, Rome, Italy)) and Group B (test) (CHX gel (Plak-Gel; Polifarma Wellness Srl, Rome, Italy)) using computer-generated random numbers, centralized with sealed opaque envelopes provided in sequence by the study consultant. Patients were informed of all study procedures but blinded to the different gels used in the study.

### 2.2. Sample Size and Randomization

The MBL was used to assess the number of patients to be randomized. Following the publication by Annibali et al., 2012 [25], the number of patients per group (15) was calculated as the sample size to have a minimum difference in MBL between the two groups of −0.55 mm at follow-up with an expected standard deviation of 0.5 mm. The value of α was determined to be 0.05, while the test power was 0.80. The Pass 3 software and the two-sample *t*-test with equal variance were used for the calculation. The number of patients were increased by 20% to offset patient loss at follow-up that could invalidate the test. Eighteen patients were then selected for each group.

### 2.3. Surgical and Prosthetic Treatment

During the initial evaluation, all subjects underwent clinical and radiographic examinations before scheduling their surgical procedures. Throughout all stages, a gel containing 0.20% CHX (Plak-Gel; Polifarma Wellness Srl, Rome, Italy) in Group B or a placebo gel (Placebo, Polifarma Wellness Srl, Rome, Italy) in Group A was applied inside the connection. The gels were indistinguishable in packaging, color, and odor. The identity of the gels was only disclosed after data collection by the gel-preparer. Prior to the procedure, patients rinsed their mouths with a 0.2% chlorhexidine digluconate solution for 2 min to decrease the bacterial load. All patients received a 2 g/day antibiotic therapy for 6 days (Augmentin; GlaxoSmithKline Beecham, Brentford, UK) along with postoperative instructions. Implant insertions (Cortex classic, Cortex, Shalomi, Israel) were carried out by two qualified operators at T0. A healing abutment was positioned at 8 weeks (T1), followed by the placement of a temporary acrylic restoration at week 16 (T2). A single-crown implant-supported cemented restoration was realized using porcelain fused to metal and inserted 18–20 weeks post-implant insertion (T3). The first radiological study was performed at 12 months of follow up (T4). The long-term follow-up period was set at 5 years (T5), during which patients were encouraged to attend regular check-ups and oral hygiene sessions. In 2022, all patients were invited for a follow-up visit that included periapical X-ray evaluations of marginal bone loss, and full mouth plaque and bleeding scores, as well as recording any mechanical complications and/or failures as better explained in the Section 2.4.

### 2.4. Patient Analysis

Implant success was assessed based on the clinical and radiographic criteria established by Papaspyridakos et al., 2012 [26]. The data were collected in patient-specific Clinical Record Forms (CRFs). Peri-implant and gingival indices, including Full Mouth Plaque Score (FMPS) and Full Mouth Bleeding Score (FMBS), were recorded at every stage of the study. As thoroughly explained in [23], radiographs and clinical records were consistently taken throughout the study. To compare radiographic changes in the marginal peri-implant bone, an analogical intraoral radiograph was taken, processed on digital software for scientific evaluations with an accuracy of less than 0.1 mm, and the mean value between the mesial and distal region was used for analysis.

Customized commercially available Rinn film holders were employed for each subject to achieve a highly reproducible and accurate image. Radiographs were repeated at each time point, and follow-up analyses were also recorded at the 5-year mark (T5). In each radiograph, the distances from the fixture’s apex to the mesial and distal crestal bone levels at the first bone–implant contact were measured. Additionally, the length and diameter of the implants were gauged to ensure accurate measurement, even if the implant appeared slightly angled on the radiograph. Based on these measurements, a computerized calibration was executed, and linear measurements of Marginal Bone Loss (MBL) were determined using ImageJ 1.48 (Bethesda, MD, USA).

At follow up visits, one examiner recorded and analyzed the following information:

- Periapical X-ray;
- FMPS and FMBS;
- Frequency of follow-up visits.

Moreover, any mechanical complications were recorded to provide a more comprehensive overview of the peri-implant health of the treated patients.

### 2.5. Statistical Analysis

Statistical tests were conducted using Excel (Microsoft, Redmond, WA, USA) and GraphPad 8 software (San Diego, CA, USA). The statistical tests to be used were predetermined by the study protocol. Patients were included in the statistical evaluation, and data are presented with means and standard deviations (SD). Analysis of variance (Student’s *t*-test) was used to assess differences between groups, at the 5 different time points considered in the study. Significance was set at *p* = 0.05.

## 3. Results

Initially, 40 patients underwent screening to determine their eligibility based on inclusion and exclusion criteria. Initially, 34 patients were recruited for single-implant-supported restorations. Six patients were deemed ineligible due to non-compliance with the inclusion criteria, and an additional two were excluded post-randomization due to inadequate oral hygiene at the surgical appointment. Consequently, a total of 32 patients were included in the initial study for single-implant restorations. Of these, 31 patients remained eligible for the 5-year follow-up evaluation, with one patient excluded due to relocation away from the clinic.

Of the remaining 31 patients, 16 patients were in Group A (placebo gel control group) and 15 patients were in Group B (chlorhexidine gel).

**Figure 1:**
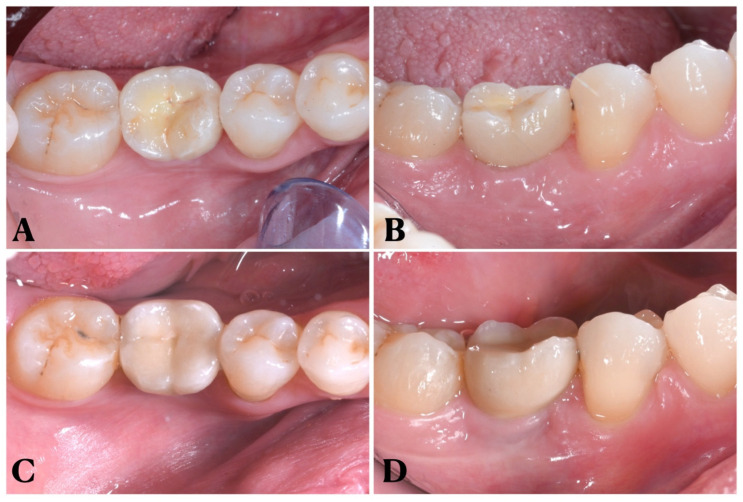
Clinical images at different time points: (**A**,**B**) occlusal and vestibular view at T4 (12-month follow-up); (**C**,**D**) occlusal and vestibular view of same patients at T5 (5-year follow-up).

Figure 1 shows intraoral clinical photos at two different time points, T4 (12-month follow-up) and T5 (5-year follow-up). The clinical cases depicted here are the same ones featured in the previous paper, which examined patients up to the 12-month follow-up [23].

Among the analyzed patients, only one implant failure was recorded (belonging to Group A), demonstrating a total success rate of 96.7% at 5 years. At 12 months, the success rate was 100%. The patient with implant failure complained of pain and mobility during the T5 follow-up visit. At first, prosthetic screw loosening was suspected. During the appointment, however, the implant was removed while removing the restoration. Except for the patient who had implant failure, all other restorations were in good general health. No patient reported pain, paresthesia, or other biological complications related to implant placement. The data regarding the enrolled patients are shown on Table 1.

Of the controlled patients, 18 attended follow-up visits and oral hygiene sessions over the years. The remaining 13 unfortunately did not attend maintenance sessions and follow-up visits. That is, 41.9% of patients were out of the control of the dentist. At T5, periodontal indices were collected for all patients who were then subjected to rigorous periodontal hygiene control. The periodontal indices recorded had an overall mean value of less than 25%. However, by dividing the various groups, different scores were obtained. No statistically significant difference was found between Groups A and B. On the contrary, a statistically significant difference (*p* < 0.05) was found between the group receiving hygiene maintenance and the group that did not, as shown in Table 1.

**Figure 2:**
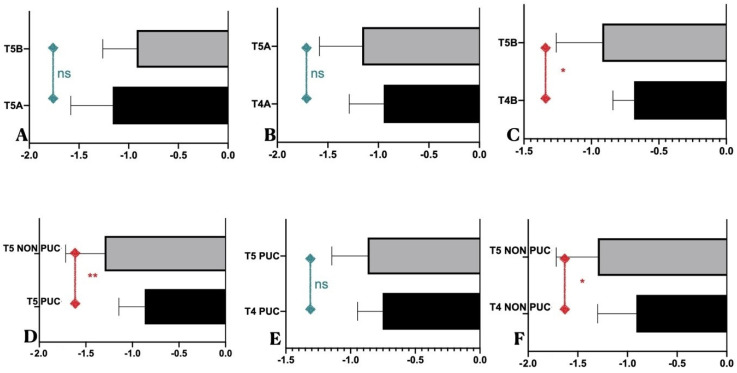
Statistical analysis of MBL: (**A**) MBL comparison at T5 between patients of Groups A and B. No statistical differences were found at 5-year follow up; (**B**) MBL comparison between T4 and T5 in patients belonging to Group A. No statistical differences were found between these two time points. (**C**) MBL comparison between T4 and T5 in patients belonging to Group B. A slight statistical difference was found between these two time points; * *p* ≤ 0.05. (**D**) MBL comparison at T5 between patients under or not under periodontal control (PUC vs. NO-PUC). A statistical difference was found in MBL between patients under and not under control; ** *p* ≤ 0.001. (**E**) MBL comparison between patients under periodontal control (PUC) between T4 and T5. No statistical differences were found between these two time points; (**F**) MBL comparison between patients that did not undergo at control visit (NO-PUC), at T4 vs. T5. A statistical difference was found in MBL in patients under control; * *p* ≤ 0.05.

**Figure 3:**
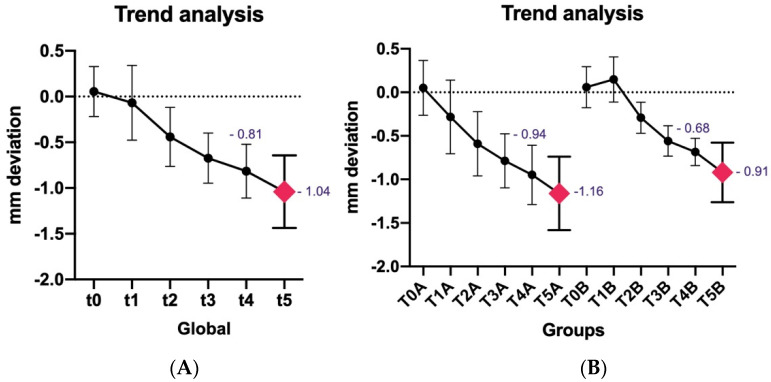
Graphical representation of MBL during every surgical and prosthetic stage until T5. (**A**) Global MBL; (**B**) MBL in Group A vs. Group B.

**Figure 4:**
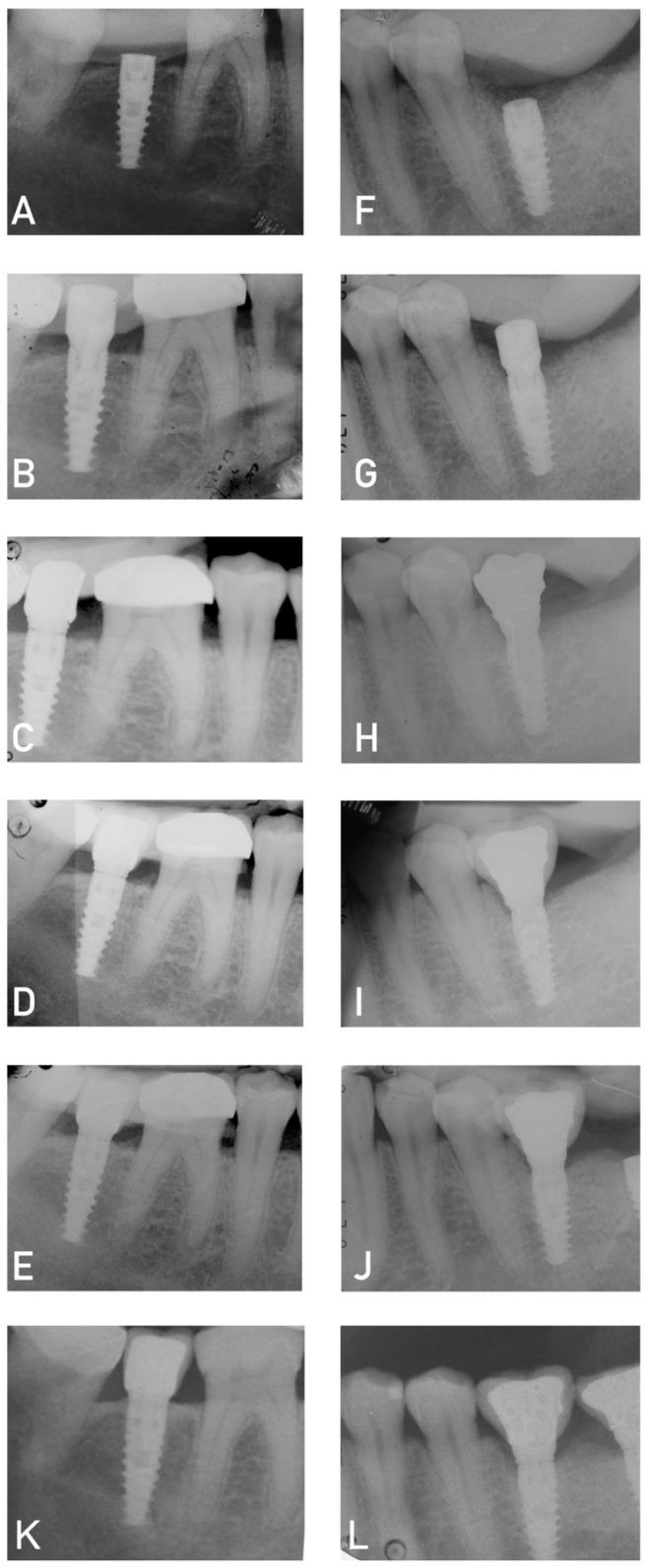
Radiographs from the two groups. (**A**–**E**) Patient from Group B (test group) from T0 to T4. A minimal bone gain was present at the second surgical stage. (**F**–**J**) Patient from Group A (control group); (**K**) radiograph of same patient from Group A at T5. No bone loss was present at 5-year follow-up; (**L**) radiograph of same patient from Group A at T5. A slight bone loss was present at 5-year follow-up.

The collected radiographs allowed the monitoring of bone resorption over the 5 years. A global bone loss of 1.04 ± 0.39 mm was recorded. However, different analyses have been proposed to understand the trend over the years. The MBLs were recorded and divided by group membership as shown in Table 2 and Figure 2 and Figure 3, with no statistically significant differences observed between the groups. Figure 4 show different MBL between the two groups during the different stages from T0 to T5. A statistically significant difference was detected when we compared patients under annually periodontal control and patients that did not attend annual control visits or undergo professional oral hygiene treatment.

**Figure 5:**
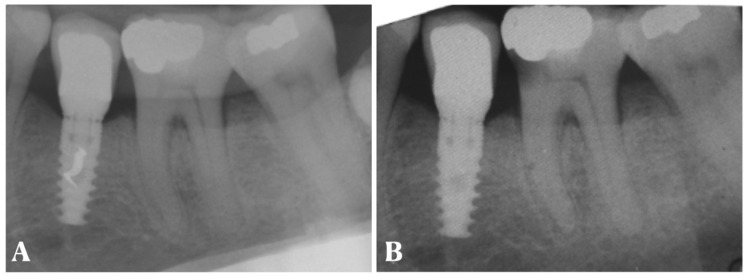
Periapical radiographs: (**A**) T4 (12-month follow-up); (**B**) T5 (5-year follow-up). The patient did not attend the control visits. A caries was identified under the old restoration in the mesial portion of the adjacent tooth.

In addition, several mechanical complications were recorded over time (loosening of the screw, chipping of the ceramic or fracture, and decementation of the crown). Specifically, seven crowns (22.5%) showed screw loosening over the years, despite being tightened according to the protocol suggested by the company. In five cases, the unscrewing occurred multiple times (16.1%). Other minor complications recorded were crown decementation over the years (three cases, 9.6%), no case of ceramic coating fracture and two cases of chipping (6.4% of cases). In a single case, a caries was recorded on the mesial element of the implant restoration in a patient who had never presented for annual hygiene maintenance sessions over the years. The Rx is shown in Figure 5. No major mechanical complications (abutment or implant fracture) were recorded.

## 4. Discussion

The aim of the present study was to demonstrate the potential efficacy of chlorhexidine in maintaining single-implant-supported rehabilitations. However, the data showed no statistically significant difference between the test group and the control group in terms of the primary objective. Therefore, the null hypothesis is accepted, suggesting that CHX has no influence on MBL after five years of follow-up. However, the secondary objectives yielded intriguing results with statistically significant differences, as explained below.

The patient group was previously evaluated after 1 year of loading, where a 100% survival rate was reported. Within the cohort of patients who completed the follow-up assessment, one implant was extracted as a result of failure, resulting in an overall success rate of 96.7% after 5 years. The overall 5-year outcome is consistent with studies on other implant systems [27,28,29]. For instance, Doornewaard et al. reported a 5-year survival rate of 97.3%, independent of the implant surface [29].

However, the data analysis has revealed interesting differences in the 5-year follow-up controls. Firstly, global MBL demonstrated slight bone resorption in both groups, with a global bone loss of 1.04 ± 0.39 mm. It is known that these data are strongly influenced by numerous factors such as implant design, the patient, clinical procedures, and the design of the implant neck [8]. However, bone loss is expected during the first year of function due to remodeling and adaptation [8,11,23]. Other authors have evaluated the progression of bone resorption in different cohorts [30,31,32]. For example, Zumstein et al. in 2019 assessed the MBL trend in a specific implant morphology in patients treated with or without GBR procedures [30]. The results showed no statistically significant differences between the groups, with a global MBL of 0.7 ± 0.7 mm after 1 year and 0.8 ± 0.6 mm after 5 years, in line with the results presented here. However, different factors influenced bone remodeling in Zumstein et al.’s 2019 study, including age, gender, implant position, biotype, implant diameter, implant length, indication, surgical/loading protocol, and ISQ at prosthesis delivery [31]. The data presented in this study indicate that the utilization of CHX did not exert a discernible impact on marginal bone loss (MBL) at the 5-year follow-up. Nevertheless, it is noteworthy that these same patients exhibited statistically significant disparities in MBL at the 12-month assessment [23]. Specifically, at T4 (12-month follow-up), patients treated with CHX showed an MBL of 0.68 ± 0.15 mm compared to 0.94 ± 0.34 mm in the control group. In the same patients, an MBL of 0.91 ± 0.33 mm (CHX) and 1.16 ± 0.42 mm (control) were present at the 5-year follow-up. No statistically significant difference was present between the two groups at 5 years, but an advantage in terms of MBL was preserved in the CHX group at the 5-year follow-up. Trend analysis established the maintenance of this advantage in all patients treated with CHX. Zumstein et al. in 2019 analyzed MBL among different factors, confirming that the trend of MBL remains unchanged from 1 to 5 years depending on the analyzed criteria. Age, implant position, and gingival biotype showed statistically significant MBL differences. The groups that showed an advantage at 1 year retained it at the 5-year follow-up [31]. Similarly, patients treated with CHX maintained an advantage in terms of MBL even at 5 years. These data illustrate that early management of risk factors, encompassing decontamination of the implant connection with CHX, facilitates more efficient maintenance and attenuates long-term bone remodeling.

The effects of CHX have also been extensively investigated in the surgical treatment of peri-implantitis [19,22]. However, its benefits are limited due to its short-term application. Some studies have shown that CHX can successfully kill bacteria in biofilms grown on titanium surfaces [21,33,34]. However, CHX seems to be only modestly effective in removing the biofilm [35]. It shows a limited killing effect related to time exposure. Therefore, the application of CHX in all early stages allowed for the control of bacterial contamination, showing a reduced MBL up to 12 months [23]. In another study by D’ercole et al. (2020) on the same patients, microbiological and immunohistochemical investigations demonstrated a reduction in inflammatory responses and bacterial loads in patients treated with CHX [24]. At the 5-year follow-up, the effect had disappeared, but the advantage in terms of MBL was still maintained.

MBL was also evaluated by dividing patients into those under strict periodontal control (at least one annual visit) and those not under control. In this sense, statistically significant differences emerged between the two groups, where the latter showed a statistically significantly higher MBL. Among the risk factors for implant failure, hygienic maintenance and control visits still represent a challenge. On the other hand, the incorrect use of periodontal hygiene aids can alter the surface roughness of titanium, increasing bacterial proliferation. Therefore, not only proper patient maintenance but also correct techniques and instrumentation are necessary to manage the follow-up of implant patients [36]. Several studies have indicated that individuals with periodontitis exhibit reduced implant survival rates and a heightened incidence of complications following implant restoration [37,38]. The rates of peri-implantitis in patients with periodontitis are higher than those in healthy individuals [39,40]. It is essential to emphasize that the importance of recalls and follow-up appointments to ensure oral health has been widely demonstrated, even in patients with special needs. In these patients, those under periodontal control can maintain a higher level of oral health, ensuring a more favorable prognosis [41]. However, other studies proposed that, under the condition of strictly controlled periodontitis, the implant survival rate could also be at a high level [42,43,44,45]. Patients with poor periodontal hygiene control not only showed a worsening of MBL but also a higher presence of gingival indices and other complications compared to patients under strict control. The recorded periodontal indices (plaque index and bleeding index) had an overall mean value of less than 25%. However, a statistically significant difference (*p* < 0.05) was found between the group receiving hygiene maintenance and the group that did not. Different data have emerge in the literature on this topic. Some studies show implant loss rates higher than 15% in untreated periodontitis patients. On the other hand, comparable survival rates have been demonstrated in patients with periodontitis under strict periodontal control [31]. These data demonstrate that, despite the decontamination of the connection made with CHX, poor hygiene control promoted worsening in terms of gingival indices and MBL over time, nullifying any beneficial effects of the initial CHX treatment.

Furthermore, the height of peri-implant soft tissues can influence the choice of transmucosal pathway height, bone remodeling, and patient hygiene maintenance [46]. In our treated patients, the implant system allowed for the systematic selection of various abutment heights based on clinical considerations. Following an assessment of soft tissue thickness, the most suitable abutment height was chosen for each individual patient, positioning it approximately 1 mm below the level of the soft tissues. Consequently, despite variations in abutment heights, all crowns were fabricated to exhibit optimal emergence profiles, facilitating effective patient hygiene maintenance. It is important to bear in mind that in addition to controlling biological complications (gingival indices, MBL, and survival rate), hygiene control also helps to control mechanical complications and the onset of any additional problems. Among the patients with poor hygiene control, one had secondary caries beneath an old restoration on a tooth adjacent to the implant. Among the mechanical complications (22.5%), loosening was experienced over the years, despite being tightened according to the protocol suggested by the company. Screw loosening is a commonly observed implant complication [47,48,49]. Jemt et al. reported that screw loosening occurred in 27.3% of 107 single-implant restorations placed in more than 90 patients [48]. For 5 years, Kreissl et al. observed partially edentulous patients who had undergone implant treatment and reported screw loosening in 6.7% of cases [49]. Cho et al. observed 213 dental implant patients over a period of 3 to 7 years and reported that screw loosening occurred in 10.3% of single-implant restoration cases and 12.1% of multiple-implant restorations [50]. Screw loosening was observed in 7.2% of the implants, typically occurring once (77.7%), followed by twice (14.4%), and more than twice (7.9%). Most cases occurred within six months of loading (50.4%). The data vary depending on implant diameter and restoration type (screw-retained or cement-retained) [47]. In our patients, 22.5% presented screw loosening, with 16.1% experiencing multiple loosening episodes. These data confirm that abutment loosening remains the primary mechanical complication in cement-retained restorations. Ceramic chipping was present in 6.4% of cases, and crown decementation in 9.6%, indicating a lower incidence than decementation.

It is important to recognize the primary limitation of this study. The assessment of MBL through intraoral radiography may be influenced by variations in cortical bone density and morphology. However, to mitigate this potential bias, all radiographs were consistently taken, processed, and measured following a rigorous protocol. Thus, intraoral radiography remains the preferred and most appropriate tool currently available for MBL measurement. Overall, the data demonstrate good survival of single-implant-supported restorations, with minor bone loss demonstrated between 1 and 5 years of follow-up, particularly when associated with good periodontal hygiene control. Similar results were found in previous clinical studies of two-piece dental implants with a horizontal offset, which showed only minor changes over time and bone levels close to the implant shoulder [47]. However, the bone loss in the present study was within the regular range for this type of implant, mostly occurring within the first year after loading, especially in the group that did not receive CHX treatment.

## 5. Conclusions

At 5 years of follow-up, CHX had certainly lost its effect, as both groups experienced moderate bone loss. It is known from the initial published study that patients treated with CHX during all surgical and prosthetic phases had reduced MBL at 12 months. The same patients also showed a reduced MBL at 5 years in absolute terms. It is therefore possible to hypothesize that better short-term management has beneficial effects even after 5 years of follow-up. For this reason, it could be interesting to conduct further studies to evaluate a method for administering CHX over an extended period, using hydrogels as molecules to deliver CHX in the peri-implant zone [51]. De Cremer et al., in 2017 [52], provide an in vitro proof of concept of the sustained release of chlorhexidine from Ti/SiO_2_ materials thereby preventing and eradicating biofilm formation on the surface of the dental implant. These types of investigations should be expanded to better understand the potential of sustained release of CHX.

Finally, in patients with poor periodontal hygiene control, MBL and periodontal indices are statistically worse. Periodontal hygiene control remains a fundamental aspect in the management of implant patients as a tool to intercept biological and mechanical problems with the implants or adjacent teeth.

## Figures and Tables

**Table 1:** Patient characteristics at T5. Patient number, survival rate, gingival indices, and statistical analysis of FMPS and FMBS. PUC = patients under periodontal control. NO-PUC = patients that did not undergo the periodontal control. Sig. **** (*p* ≤ 0.0001), *** (*p* ≤ 0.001), ns (non-statistically significant).

Total patient number	32		
Patients evaluated at 5-year follow-up	31		
Patients in Group A	16		
Patients in Group B	15		
Implant failures	1 (Group A) implant removed		
Survival rate	96.7%		
Patients under strict hygienic control (PUC)	58.1%		
Global Full-Mouth Plaque Score (FMPS)	21.38% ± 5.65		
Global Full-Mouth Bleeding Score (FMBS)	20.96% ± 4.76		
	Group A	Group B	Sig.
FMPS (Group A–Group B)	22.59% ± 5.93	19.83% ± 5.22	*p* = 0.18 ns
FMBS (Group A–Group B)	21.37% ± 5.46	20.39% ± 4.23	*p* = 0.59 ns
FMPS (PUC/NO-PUC)	17.81% ± 3.37	26.32% ± 4.42	****
FMBS (PUC/NO-PUC)	18.17% ± 2.88	24.95% ± 4.3	***

**Table 2:** Table show all the patients. Specifically, they were divided in Group A (control) and B (CHX gel). PUC = patients under periodontal control, NO-PUC = patients that did not undergo the periodontal control. All MBL were reported at T0 to T5 and FMPS and FMBS at T5 were reported. Patient ID34: the implant was removed.

ID PAT	SITE	GROUP	PUC/ NO-PUC	T0	T1	T2	T3	T4	T5	FMPS	FMBS
2	16	A	NO-PUC	0.22	−0.06	−0.49	−0.73	−1.51	−1.6	22.4	24.5
5	14	A	PUC	0	−0.38	−0.55	−0.82	−1.07	−1.1	21.3	19.45
7	46	A	PUC	0.49	0.12	−0.2	−0.84	−0.95	−1.2	24.5	17.54
11	16	A	NO-PUC	−0.62	−1.53	−1.75	−1.76	−1.84	−2.2	25.6	18.34
14	47	A	PUC	−0.39	−0.92	−0.89	−0.65	−0.8	−0.87	19.3	18.5
17	36	A	NO-PUC	−0.06	−0.38	−0.43	−0.68	−0.73	−0.88	29.4	28.76
20	36	A	PUC	0.06	0	−0.44	−0.6	−0.63	−0.7	16.6	19.5
21	46	A	PUC	0.16	−0.29	−0.53	−0.73	−0.94	−0.9	14.3	14.5
23	46	A	NO-PUC	−0.13	−0.2	−0.74	−1.01	−1.05	−1.68	31.4	29.04
24	35	A	NO-PUC	−0.06	−0.2	−0.44	−0.59	−0.64	−1.5	30.7	31.84
25	22	A	PUC	0.6	−0.06	−0.46	−0.71	−0.77	−0.82	24.3	19.12
29	36	A	PUC	−0.11	−0.15	−0.78	−0.94	−1.06	−1.25	17.5	18.76
30	37	A	PUC	0.05	−0.09	−0.25	−0.38	−0.58	−0.87	15.4	14.45
32	24	A	NO-PUC	0.35	−0.05	−0.52	−0.79	−0.9	−1.1	29.87	27.12
33	36	A	PUC	0.21	−0.05	−0.4	−0.58	−0.74	−0.74	16.3	19.09
34	46	A	NO-PUC	−0.08	−0.21	−0.58	−0.61	−0.8	REMOVED	26.5	23.45
1	36	B	NO-PUC	0.57	0.62	0	−0.47	−0.55	−1	25.8	26.09
4	36	B	PUC	−0.17	0.03	−0.5	−0.71	−0.81	−1.32	14.6	21.45
8	46	B	NO-PUC	0.2	0.21	−0.48	−0.74	−0.79	−1.2	29.45	24.59
10	36	B	PUC	−0.04	0	−0.38	−0.53	−0.61	−0.6	20.5	19.4
12	24	B	PUC	0	0.1	−0.35	−0.55	−0.71	−1.1	20.4	22.22
13	36	B	NO-PUC	−0.02	0.15	−0.37	−0.67	−0.89	−1.25	19.3	18.41
15	36	B	NO-PUC	0.16	0.39	−0.08	−0.61	−0.74	−1.45	17.9	19.45
16	46	B	PUC	0.12	0.59	−0.23	−0.49	−0.61	−0.72	15.9	18.54
18	15	B	PUC	0	−0.36	−0.57	−0.69	−0.77	−0.8	14.1	13.4
19	46	B	PUC	0.34	0.24	0	−0.16	−0.31	−0.56	14.5	12.23
22	24	B	PUC	0.29	0.28	−0.29	−0.88	−0.95	−0.23	14.5	16.98
26	45	B	PUC	−0.35	−0.08	−0.15	−0.41	−0.66	−1.12	16.4	19.5
27	37	B	NO-PUC	0	−0.05	−0.21	−0.54	−0.59	−0.62	27.8	24.98
28	25	B	PUC	0.06	0.18	−0.3	−0.37	−0.57	−0.72	20.12	22.45
31	26	B	NO-PUC	−0.26	−0.08	−0.46	−0.56	−0.7	−1.1	26.23	26.23
							MEAN	−0.8151613 mm	−1.04 mm	21.38%	20.96 %
							ST. Dev	0.28814199	0.39673669	5.65255588	4.76767566

## Data Availability

All data generated or analyzed during this study are included in this published article. Trial registration: Trial is registered with ClinicalTrials.gov (Registration Number: NCT03431766). Registered 13 February 2018 (retrospectively registered).
